# Integration of Multiple Signaling Pathways Determines Differences in the Osteogenic Potential and Tissue Regeneration of Neural Crest-Derived and Mesoderm-Derived Calvarial Bones

**DOI:** 10.3390/ijms14035978

**Published:** 2013-03-15

**Authors:** Kshemendra Senarath-Yapa, Shuli Li, Nathaniel P. Meyer, Michael T. Longaker, Natalina Quarto

**Affiliations:** 1 Hagey Laboratory for Pediatric Regenerative Medicine, Department of Surgery, Stanford University, School of Medicine, Stanford, CA 94305, USA; E-Mails: kshem@stanford.edu (K.S.-Y.); shuli@stanford.edu (S.L.); nate.meyer6@gmail.com (N.P.M.); 2 Department of Advanced Biomedical Science, University of Studies of Naples Federico II, Naples 80131, Italy

**Keywords:** neural-crest, paraxial-mesoderm, origin, bone, regeneration, signaling, apoptosis

## Abstract

The mammalian skull vault, a product of a unique and tightly regulated evolutionary process, in which components of disparate embryonic origin are integrated, is an elegant model with which to study osteoblast biology. Our laboratory has demonstrated that this distinct embryonic origin of frontal and parietal bones confer differences in embryonic and postnatal osteogenic potential and skeletal regenerative capacity, with frontal neural crest derived osteoblasts benefitting from greater osteogenic potential. We outline how this model has been used to elucidate some of the molecular mechanisms which underlie these differences and place these findings into the context of our current understanding of the key, highly conserved, pathways which govern the osteoblast lineage including FGF, BMP, Wnt and TGFβ signaling. Furthermore, we explore recent studies which have provided a tantalizing insight into way these pathways interact, with evidence accumulating for certain transcription factors, such as Runx2, acting as a nexus for cross-talk.

## 1. Introduction

Mammalian calvarial development and homeostasis are tightly regulated processes, dependent on the interplay of osteoblasts and osteoclasts and orchestrated by key, highly conserved, signaling pathways. The outcome of this developmental program is the mammalian skull vault, which itself can be regarded as the product of an evolutionary process during which four skeletal components of independent origin have been progressively integrated into a structure of exquisite structural and functional complexity [1]. Much progress has been made in recent years in defining the multiple signaling pathways, which confer osteogenic potential and regenerative capacity on the embryologically disparate calvarial bones. This is accomplished through regulation of the osteoblast lineage in terms of function, proliferation, commitment, differentiation, maintenance of an undifferentiated progenitor pool and interestingly also on apoptosis, the process of programmed cell death. In this review we will explain how it has been possible to progress from a deeper understanding of the embryonic origin of the mammalian skull vault to rigorous *in vivo* and *in vitro* analysis of the differences in activity of key signaling pathways between the neural crest-derived frontal bones and the paraxial mesoderm-derived parietal bones. Furthermore, given the pivotal role played by neural crest cells in conferring increased osteogenic potential and regenerative capacity on frontal bones and in establishing the regional differences that we have observed, a brief outline of their development and biology will be provided. We will place the work of our laboratory in studying this elegant model of regional embryonic differences into the wider context of our current understanding of the roles played by these ubiquitous and highly conserved pathways. Finally, we will discuss how this work has provided novel insights into the way these pathways interact with each other to govern osteoblast behavior and thereby bestow osteogenic potential and regenerative capacity on calvarial bones. Ultimately, as we move towards a more comprehensive understanding of the regulation of osteoblast behavior through this incremental approach, it is envisaged that it will be possible to identify the most suitable targets in this signaling network for selective pharmacological modulation in order to enhance endogenous skeletal regenerative capacity and potentially deliver significant translational benefit in craniofacial reconstruction.

## 2. Development of the Mammalian Calvarium: A Model to Study the Integration of Multiple Signaling Pathways

The four skeletal components of the vertebrate skull are the cartilaginous neurocranium, cartilaginous viscerocranium, dermal skull roof and the sclerotomal occipital region [1]. Osteoblasts can be produced from mesenchymal stem cells by two distinct processes during vertebrate embryogenesis: intramembraneous and endochondral ossification [2,3]. The dermal skull roof, which is evolutionarily derived from the protective dermal plates of early jawless fishes, is formed from the more ancient process of intramembraneous ossification in which mesenchymal progenitors condense and subsequently differentiate directly into osteoblasts while endochondral ossification, which principally plays a role in the axial skeleton, occurs via the formation of a cartilaginous intermediate [4]. Importantly, the five principle bones of the mammalian skull vault which includes the paired frontal bones, the paired parietal bones and the unpaired interparietal bones arise from two distinct embryonic origins; neural crest cells which are a mesenchymal cell type from the neural ectoderm unique to vertebrates [5], and the paraxial mesoderm. Historically there has been considerable debate regarding the disparate embryonic origin of calvarial bones, specifically the frontal and parietal bones. Early studies extrapolated data from avian models because of difficulties at the time with cell and tissue lineage studies in mammalian embryos and drew different conclusions as to their embryonic origins [6–8].

**Figure 1:**
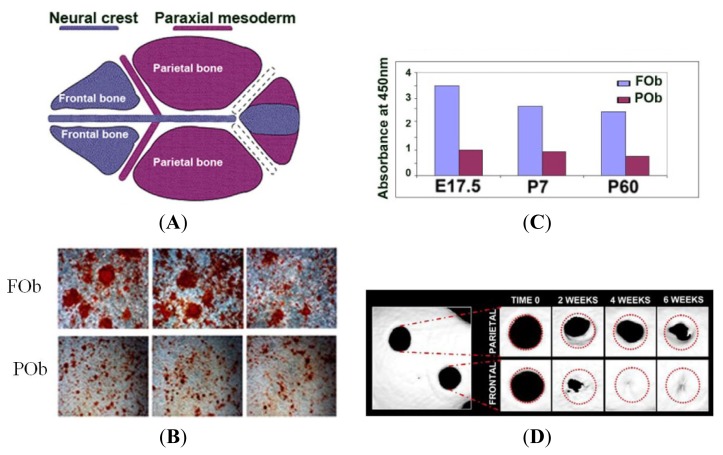
(**A**) cartoon depicting neural crest and paraxial mesoderm origin of the cranial; (**B**) alizarin red staining of frontal (FOb) and parietal (POb) bone-derived osteoblast cells at osteogenic differentiation day 21 shows striking differences between FOb and POb, with FOb cells having a more robust mineralization and larger bone nodules; (**C**) quantification of alizarin red staining; (**D**) Micro-computed tomography (μCT) up to 8 weeks after creation of 2 mm calvarial defect in frontal neural crest-derived and paraxial mesoderm-derived parietal bones revealed significantly increased healing of frontal bone defect compared to parietal bone in P7 mice. Abbreviations: (**E**), embryonic; (**P**), postnatal.

Quail-chick chimera studies performed by Noden *et al.*, supported prior observations by Le Lievre that the cranial vault had a dual origin consisting of tissue derived from neural crest cells and mesoderm [6,7]. Using the same model however Couly *et al.* reported contradictory findings that the skeletal tissue of the cranial vault consisted solely of neural crest cells [8]. These quail-chick chimera studies were, however, blighted by several constraints including the fact that the skull vault bones had only just begun to mineralize at the time the experiments were concluded at E14, and because of poor delineation of the calvarial sutures at this stage, the small size of the avian parietal bones, and the absence of postparietal bones. These deficiencies therefore, as Moriss-Kay noted, may have contributed to the disparities in the interpretation of the data gained through the study of this model, and the conclusions drawn thereafter [1]. Extrapolations form avian data must also be drawn with some caution given the early evolutionary divergence of birds and mammals from reptilian lines, from which they arose, and the clear anatomical differences in the skull roof patterns in these groups [1]. The real paradigm shift in our understanding of the embryonic origin of the mammalian skull vault dawned with the arrival of transgenic mouse technology. Jiang *et al*., using mice with the *Wnt1-Cre* transgene, which is expressed solely in neural crest cells, with the conditional *LacZ* reporter, R26R, which is only expressed when activated by Cre, showed conclusively that the frontal and squamosal bones are neural crest derived, whereas the parietal bones are of mesodermal origin [9]. More recently, Yoshida *et al.* elegantly verified the same disparate dual embryonic origin of the frontal and parietal bones, by conducting the reciprocal study using the *Mesp1-Cre* transgene combined with R26R, which specifically and permanently labelled mesodermal cells [10]. In this way, they were able to validate their previous work, which relied solely on *Wnt-1*, a permanent cell marker for neural crest cells [9,10]. Given that the mixed developmental origin of the mammalian skull vault had therefore been established, we wanted to investigate the impact that this unique developmental and evolutionary history had on the molecular and genetic control of cell and tissue interactions and in particular, the effect on calvarial healing in neural crest derived frontal and paraxial mesoderm derived parietal bones. Encouraged by our early studies using both *in vitro* osteogenic differentiation assays and a mouse calvarial defect model, which clearly demonstrated the superior osteogenic potential and healing capacity of neural crest-derived frontal bones and their derived osteoblasts [11] (Figure 1), we sought to use this calvarial model to elucidate, in a step-wise fashion, the molecular signaling pathways which confer superior osteogenic potential and regenerative capacity on frontal bones. We will describe how this approach has provided novel insights into the multiple signaling pathways that determine osteogenic potential and how they may communicate with each other to coordinate this process. Moreover, this work will be placed into the wider context of our current understanding of these signaling pathways as the scientific community moves closer to a deeper understanding of a core signaling network which orchestrates the osteoblast lineage.

## 3. The Neural Crest

In the context of bone biology and, in particular, in the context of our aims to explore the impact of mammalian calvarial embryogenesis on conferring different regenerative capacities on embryologically disparate calvarial bones, the neural crest deserves a special mention. The Neural Crest (NC) is a transitory structure, which forms from the lateral borders of the neural plate as they join in the midline during closure of the neural tube [12]. It is a remarkable population of multipotent embryonic cells unique to vertebrates, which migrate from the dorsal neural tube early in development to give rise to a diverse array of derivatives, including cardiac cells, melanocyte, neurons and glia of the peripheral nervous system, and most of the bone and cartilage of the face and skull [12,13].

The formation of NC has been described as a classic example of embryonic induction, in which specific tissue interactions and the concerted action of signaling pathways converge to induce a multipotent population of neural crest precursor cells. The process of NC induction is a multistep process from gastrulation to neurulation. In the first phase NC formation is initiated by several environmental signals eliciting their effects on cells at the neural plate border. This involves the combinatorial input of multiple signaling pathways, among them BMP, Wnt, FGF and Notch. During the second phase BMP, Wnt, and Notch signaling maintain these NC-progenitor cells and bring about the expression of definitive NC markers including Snail2, FoxD3, and Sox9/10 [12–14].

The neural crest cells are mesenchymal cells derived from the neural crest epithelium. Two key features of neural crest cells are migratory ability and multipotency [12,13,15]. The earliest migrating cells populate the facial processes and give rise to mesenchymal derivatives, while later migrating NC cells remain in more dorsal regions and contribute to cranial ganglia. Depending on the site of origin along the anterior-posterior axis of the embryo, neural crest cells fall into 3 populations, cranial, cardiac, and trunk, each with a unique developmental potential. The cranial neural crest (CNC), which originates in the portion of the neural tube from the neural fold anterior to rhombomere 6 give rise to much of the cartilage and bone of the skull and face, as well as other connective tissues, and contribute to neurons and glia in cranial ganglia. Trunk neural crest cells form a more limited set of cell types, including peripheral nerves, melanocytes, and the adrenal medulla. Cardiac neural crest cells contribute to the smooth muscle lining of the outflow tract.

Great interest in NC cells has been generated by investigations on the multipotency of these cells. A recent work by Ishii *et al*. described the isolation of CNC cells with sustained stem-like potency, ability to grown as multipotent stem-like cells and expression of the stem cell marker CD44 [16]. These cells can be propagated and passaged indefinitely, and can differentiate into osteoblasts, chondrocytes, smooth muscle cells, and glial cells.

Furthermore, as NC cells bear the key hallmarks of stem and progenitor cells, they also carry potential for the development of novel applications in cell-based tissue and disease-specific repair.

## 4. The Key Signaling Pathways and How They Achieve Coordination through Cross-Talk

### 4.1. The FGF Signaling Pathway

Fibroblast Growth Factors (FGFs) constitute a family of polypeptides, which play key roles in a range of vital cellular and developmental processes. In particular, FGF signaling has been the subject of great attention for its role in prenatal and post-natal skeletogenesis including calvarial osteogenesis [17–23]. FGF signaling is also known to play a role in proliferation and differentiation of osteoblasts [24–27]. FGFs bind to high affinity FGF receptors (FGFRs) which leads to receptor dimerization, autophosphorylation of tyrosine residues and activation of the three key intracellular transduction pathways of mitogen-activated protein-kinase (MAPK, ERKs, p38 and JNKs), Protein Kinase C (PKC) and phosphoinositide 3-kinases (PI3K) [23,28]. Via these transduction pathways, FGFs can exert their effects on osteoblast gene regulation [29–32].

The role of FGF signaling in both physiological bone formation and pathological osteogenesis, as demonstrated in craniosynostosis syndromes, has been established. Iseki *et al*. demonstrated the role played by FGFR-1 and FGFR-2 in regulating the transition between osteogenic proliferation and differentiation using *in situ* hybridization on osteoprogenitor cells [33]. The transition from proliferation of progenitors to differentiation was marked by an increase in *Fgfr-1* expression and a reciprocal decrease in *Fgfr-2* expression. Gain of function FGFR mutations in humans, are associated with craniosynostosis [34]. FGF2 mutations which cause craniosynostosis have been shown to promote osteoblast differentiation via increased expression of the transcription factor *Runx2*[35], while more recently studies on FGFR-2 gain of function mutations with constitutive activation in both human and mouse osteoblasts promoted bone formation and osteoblast gene expression [36,37].

The molecular genetics approach focusing on FGF and FGFR using knockout models and the study of FGFR mutations in human skeletal disorders has delivered useful insights on the role of this pathway and in particular of *Fgf-2*, *−9* and *−18* in bone development and calvarial osteogenesis [38–41]. Montero *et al.* demonstrated decreased osteogenic differentiation of bone marrow stromal cells and calvarial osteoblasts in *Fgf-2* knockout mice [41] and went onto to describe decreased bone mass and an impaired rate of bone formation in these mutants. Moore *et al*., using beads coated with FGF-2 neutralizing antibodies demonstrated reduced calvarial osteogenesis in chicks [42]. FGF2 was also discovered to control cell fate decisions of mesenchymal stem cells between adipocyte and osteoblast differentiation [43]. A delay in proximal bone formation leading to rhizomelia has been described in *Fgf-9* knockout mice [40]. Liu *et al.* showed that *Fgf-18* null mice embryos at E15.5 and E16.5 had severely impaired calvarial ossification [44], while Ohbayashi *et al.* showed delayed osteogenesis of long bones, impaired proliferation and differentiation of calvarial mesenchymal cells, impaired differentiation of calvarial osteoblasts and delayed closure of the cranial sutures [38].

Given the weight of evidence for FGF signaling in skeletal development and calvarial osteogenesis, it was logical for our laboratory to first investigate FGF signaling as a potential determinant of the regional differences in osteogenic potential and regenerative capacity in calvarial bones of different embryonic origin. Firstly we showed, using gene expression analysis in this model, that *Fgf-2*, *−9* and *−18* ligand gene expression as well as their corresponding receptors *FgfR-1*, *−2* and *−3* were upregulated in frontal compared to parietal bones, and therefore that the neural crest derived frontal bones represented a more competent domain for FGF signaling [45]. Interestingly in the same study *Runx2*, an early osteogenic differentiation marker was upregulated in frontal bones [45]. Secondly, having previously demonstrated an enhanced healing capacity in neural crest derived frontal bones compared to mesoderm derived parietal bones [11]. We used a loss- and gain-of function approach to demonstrate that the higher endogenous levels of FGF-signaling in frontal bones was responsible for its enhanced healing capacity [46]. Exogenous FGF-ligands were able to overcome the inferior healing potential of parietal bones as specified by their embryonic origin and their inferior endogenous FGF signaling capacity [46]. In support of this concept was that calvarial healing was impaired in *Fgf-9^+/^*^−^ and *Fgf-18^+/^*^−^ haploinsufficient mice. Moreover, FGF-18 was identified as the most potent ligand for facilitating bone healing [46]. Finally using co-culture and conditioned media methods we performed *in vitro* studies to address how far back the observed differences between frontal (FOb) and parietal (POb) bone-derived osteoblasts can be traced, and to what extent FGF signaling is responsible for these differences [47]. Striking disparities were revealed with both an increased mitogenic ability and potential for osteogenic differentiation in FOb with more prominent differences at embryonic stages than postnatal stages. Interestingly, exogenous application of FGF-2 protein to POb was able to induce “frontal osteoblast like” behavior [47].

A number of important downstream molecular targets for FGF signaling have been identified. *Runx2*, a transcription factor and known target of this signaling pathway is upregulated by gain of function *FgfR2* mutations [48] and as described later can act as a nexus for cross-talk with several other signaling pathways. Interestingly, Runx2 can also interact directly with FGF-2 to influence osteoblast proliferation via effects on the extracellular matrix [23,49] Another transcription factor Twist1, a bHLH transcription factor, also highlights the complex and interesting ways that molecules can interact with signaling pathways to control osteogenesis and in particular with its interesting relationship with the FGF signaling pathway. Twist can influence FGF during bone formation [50] and can also interact directly with FGFR2 when contributing to premature suture fusion [51]. Further evidence for the intriguing relationship between *Fgf* and *Twist* genes has also been garnered by the work of Rice *et al.* who proposed a model of osteoblast differentiation integrating *Twist* and *Fgf* in the same pathway [52]. Furthermore, the same group identified Foxc1, a winged helix transcription factor, as potential point of cross-talk between the FGF and BMP pathway [53]. An interesting insight into the relationship between FGF, BMP and Msx1, was also provided, by analyzing mouse calvarial development using *in situ* hybridization in embryonic and postnatal stages of the sagittal suture [54].

### 4.2. The Wnt Signaling Pathway

The Wnt signaling pathway is a key regulator of cellular differentiation and of crucial importance in skeletal development, bone mass maintenance and remodeling and has therefore gained much attention from the research community [19,55–59]. Even minor perturbations in the intensity, context, or duration of signaling, has a major impact on bone biology. A comprehensive review of this ubiquitous signaling pathway, which plays a major role in a myriad of physiological and pathological phenomena, is beyond the scope of this review and instead therefore, we will focus on the core signaling pathway as it impacts on osteogenesis and also instances where it highlights novel methods of interaction with other key signaling pathways. For more comprehensive reviews on this broad subject please refer to recent reviews by Clevers *et al.* and Monroe *et al.*[56,59].

Wnts, a large family of secreted glycoproteins, are categorized classically according to whether they signal in a canonical β-catenin dependent or non-canonical β-catenin independent manner [60]. Canonical Wnts whose role in bone biology is better defined, act on target cells by binding to seven-span transmembrane receptors called Frizzleds (Fzds) and single span co-receptor proteins LRP-5/6 and more recently identified LRP-4 [56,59,61]. In the absence of receptor activation, β-catenin is phosphorylated at its NH_2_-terminal degradation box, which leads to ubiquitination and degradration [55]. Upon receptor activation, after binding of the appropriate Wnt ligand to the receptor complex, an intracellular protein Dishevelled (Dvl) is activated [62], which leads to the inhibition of glycogen synthase kinase 3β (GSK-3β) [63,64], preventing the degradation of β-catenin by a protein complex formed by GSK-3β, axin and adenomatous polyposis coli (APC) [64]. Upon receptor activation therefore β-catenin is not degraded, accumulates in the cytoplasm and following translocation to the nucleus through a poorly understood mechanism, acts together with members of the T cell factor/lymphoid enhancer factor (TCF/LEF) family to activate transcription of several genes [65–67].

There is significant evidence from both human and mouse gene studies implicating the canonical Wnt signaling pathway in bone development and homeostasis. Wnt/β-catenin signaling, for example, is required for the embryonic cell fate decisions of mesenchymal precursor cells in the important choice between chondrogenesis and osteogenesis. The requirement for β-catenin to repress chondrogenesis in favor of osteogenesis had been verified by loss- and gain-of-function studies. Without functioning β-catenin progenitor cells differentiated into chondrocytes [68,69]. Canonical Wnt signaling also plays a vital role in bone homeostasis by modulating the delicate balance of osteoblastic and osteoclastic activity in favor of osteoblasts and inhibiting osteoclasts [70,71]. Moreover, osteoblast specific β-catenin mutations led to osteopenia and increased numbers of osteoclasts in mice [72]. Activating mutations in the gene encoding the Wnt co-receptor LRP5 which makes it resistant to extracellular Wnt inhibitors, like Dickopff-related 1 (Dkk-1), leads to a high bone mass phenotype [73–77], whereas loss-of-function mutations in the same co-receptor leads to a juvenile form of osteoporosis, associated with decreased *de novo* bone formation, called osteoporosis-pseudoglioma syndrome [78].

Following on from our observation that disparate embryonic origin translates into regional differences in osteogenic potential and the regenerative capacity of parietal and frontal bones, and prompted by the wealth of evidence for the role of the canonical Wnt signaling pathway in skeletogenesis, we directed our attention to explore the role of Wnt signaling in determining these differences with *in vivo* and *in vitro* studies. Primary FOb cell cultures revealed a greater osteogenic potential compared to POb cells, while Micro-computed tomography following the creation of 2 mm calvarial defects in P7 and P60 mice revealed improved healing of neural crest-derived frontal bone [11]. In addition to establishing the increased endogenous Wnt signaling in FOb compared to POb, retroviral transfection of S33Y, a β-catenin mutant that accumulates in the nucleus and constitutively activates TCF mediated trancription or exogenously administered Wnt3a, bestowed a higher osteogenic potential to POb [11]. Of great interest was the finding that FGF-2 treatment could induce GSK-3β phosphorylation in POb to levels found endogenously in FOb and increase phosphorylated GSK-3β levels even further in FOb. Moreover, FGF-2 treatment also led to augmentation of nuclear levels of β-catenin. This study therefore, analyzing regional embryonic differences, provided insights into cross-talk between the FGF and Wnt signaling pathways, consistent with recent findings of many other groups. Fei *et al.* for example demonstrated that FGF-2 stimulates osteoblast differentiation in part by activating Wnt signaling [79] while Reinhold *et al.* demonstrated that *Fgf-18* is a direct target of Wnt signaling [80]. Importantly they also identified that the TCF/Lef proteins bind to a consensus target sequence of the *fgf-18* promoter and when stimulated by β-catenin induce *fgf-18* expression. Interestingly, *fgf-18* expression is also dependent on Runx2. Not only was it revealed that a recognition motif for Runx2 partially overlaps the TCF/Lef site of the *Fgf-18* promoter but also that Runx2 is necessary for stimulation of *Fgf-18* expression by Wnt, forming a ternary complex with TCF/Lef at the *Fgf-18* promoter [80]. As is described later, Runx2 may also play a role in potential interactions of the BMP signaling pathway with FGF and Wnt signaling.

### 4.3. The BMP Signaling Pathway

Bone morphogenetic proteins (BMPs), which were first purified from bovine bone and shown to induce ectopic bone formation in mice [81,82] are members of the transforming growth factor-β (TGF-β) superfamily [83]. They play a role in several biological processes [84]. They bind to heterotetramers of type I and type II Serine/Threonine kinase receptors which then phosphorylate and activate intracellular Smad proteins [85]. Receptor-Smads (R-Smads) bind to co-Smads in the cytoplasm and translocate to the nucleus where they can act as transcription factors [86]. Interestingly, an important target gene is *Runx2*. BMP2 treatment in a human marrow stroma-derived cell lines increased *Runx2* gene expression [87]. Early osteoblast differentiation was disrupted by impeding Runx2-Smad interactions following induced mutations in *Runx2*[88,89]. The role of BMPs in skeletogenesis has been demonstrated using conditional knockout alleles due to the lethality of knockout models. In this way, deletion of BMP ligands, or their receptors, from limb bud mesenchyme impairs chondrogenic or osteogenic differentiation and leads to aberrations in skeletal patterning. BMP-3, intriguingly, appears to have a different impact on skeletogenesis. It is produced by osteoblasts and osteocytes and has been shown to interact with the BMP II receptor to have an inhibitory effect [90]. BMP-3, therefore, may provide a negative feedback loop and thus demonstrate another elegant method that signaling pathways utilize to achieve temporal and spatial control.

There is much evidence for cross-talk between the BMP and other pathways in the control of osteogenesis. Several links have been made with the FGF and Wnt pathways. FGF-2 for example enhances BMP activity via inhibition of its antagonist Noggin [91] and FGF-18 represses Noggin expression [92]. Interestingly, Choi *et al*. showed that administration of FGF-2 to developing bone fronts promoted *Bmp-2* gene expression through the modulation of *Runx2* expression [93]. The relationship between BMP and Wnt signaling, however, appears to be more complex. Blocking β-catenin signaling by an adenovirus carrying Dkk-1 or a conditional floxed β-catenin reduced BMP-2 induced bone formation [94], but different mutant forms of β-catenin can antagonize or potentiate BMP-2 induced osteogenesis [95]. Indeed integration of these two pathways occurs at a number of different molecular locales, such as Runx2, β-catenin, Dvl, and GSK3β [95]. While all of these signaling pathways exert their effects through transcription factors, Runx2 deserves a special mention with regards to osteogenesis, bone development and calvarial healing. Runx2 has emerged as a master transcription factor in skeletogenesis and is necessary for osteogenesis in both endochondral and intramembraneous ossification. Homozygous deletion leads to a complete lack of differentiated osteoblasts [96,97], while haploinsuficiency of *RUNX2* in humans leads to Cleidocranial Dysplasia, which is characterized by hypoplastic clavicles and delayed closure of the fontanelles [3]. The absolute requirement for this transcription factor in osteoblastogenesis is perhaps unsurprising when one considers that it is a prime candidate as a nexus for integration of several key signaling pathways involved in this process, such as BMP, Wnt and FGF signaling.

### 4.4. The TGF-β Signaling Pathway

TGF-βs like BMPs, belong to the TGF-β superfamily of proteins and as such share commonalities in their signal transduction machinery. The R-Smads that respond to TGF-β are Smad2 and Smad3, in contrast to the Smad1 and Smad5 of BMP signaling. TGF-β signaling plays a vital role in mesenchymal stem cell and osteoblast progenitor maintenance and proliferation [98], TGF-β signaling plays an important role in both lineage selection and differentiation of almost all cell and tissue types including endochondral and intramembraneous bone. Therefore, a comprehensive account is beyond the scope of this review. We will focus on what gains we have made by analyzing the regional differences in parietal and frontal bones and highlight where important avenues for cross-talk have been identified. In addition, evidence from the TGF-β signaling pathway elegantly demonstrates the important principle of context and time dependence of signaling and how differences in these two can lead to divergent effects on bone biology. For example, the repressor versus activation function of Smad3 on Runx2 is dependent on cell context [99].

Many groups have identified potential avenues of cross-talk between the TGF-β pathway and other pathways in governing osteoblast biology. Maeda *et al.* showed that osteoblastic differentiation of mouse C2C12 cells was greatly enhanced by the TGF-β type I receptor kinase inhibitor SB431542. Endogenous TGF-β was found to induce expression of inhibitory Smads (I-Smads) during the maturation phase of osteoblastic differentiation induced by BMP-4. SB431542 suppressed endogenous TGF-β signaling and repressed the expression of I-Smads during this period, implying a possible acceleration of BMP signaling [100]. Moreover, members of TGF-β superfamily of cytokines play decisive roles in the differentiation of mesenchymal stem cells (MSCs) into osteoblasts. TGF-β promotes recruitment and proliferation of osteoprogenitors and expression of matrix proteins but inhibits late osteoblast differentiation and mineralization [101,102], while BMPs are potent regulators of osteoblast proliferation and differentiation [103]. A delicate balance exists between these two signaling pathways, which determines commitment of MSCs to differentiate toward the osteoblast lineage and the efficiency of bone formation.

Adding to growing evidence, highlighted above, for cross-talk between TGF-β and other pathways, our recent studies on embryonic stem cells and induced pluripotent stem cells (iPSCs) derived from individuals with Marfan Syndrome (MFS), who carry mutations in *FIBRILLIN 1* (*FBN1*), provided some intriguing insights into the interplay between TGF-β and BMP signaling pathways that are responsible for the skeletogenic phenotype unveiled in these cells. This phenotype features impaired osteogenic differentiation and ability to undergo chondrogenesis in a TGF-β cell-autonomous fashion [104,105]. Furthermore, these studies agreed with established literature which has demonstrated that FBN1 protein controls TGF-β bioavailability [106,107] and that enhanced TGF-β is a major causative factor for the pathology in MFS [104,108].

## 5. Integration of Multiple Pathways Controlling Apoptotic Activity in Osteoblasts

**Figure 2:**
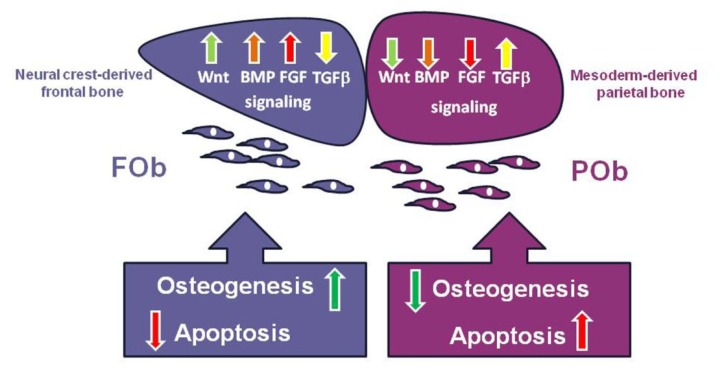
Cartoon depicting the effect of integration of differentially activated multiple signaling pathways, on neural crest-derived and mesoderm-derived calvarial bones. The differential activation of these signaling(s) promotes greater osteogenesis and less apoptosis in FOb, and conversely, less osteogenesis and higher apoptosis in POb.

Our efforts to explore and delineate the signaling which confers the different osteogenic potential and regenerative capacity on calvarial bones of disparate embryonic origin and to identify the molecular mechanism implicated in the higher osteogenic potential of frontal bone, prompted us recently to turn to another physiological process vital for skull homeostasis, apoptosis. Apoptosis, sometimes called programmed cell death, is a ubiquitous physiological process, which is important for skeletal development, tissue homeostasis, remodeling and regeneration [109–111]. Both intrinsic and extrinsic inducers can initiate apoptosis [112,113]. The balance of proliferation, differentiation and apoptosis of bone cell populations is vital for skeletal homeostasis and healing capacity as it will determine, at any one time, the balance of osteoblasts and osteoclasts. Of key importance to our investigations was that *in vitro* studies had previously shown that a higher differentiation status and bone-forming ability of osteoblasts is associated with low levels of apoptosis [114]. Additionally, excessive apoptotic activity was found to delay osteogenesis during development of mouse calvarial bone [110,115]. We, therefore, set out to investigate whether differences in apoptotic activity are present between mouse neural crest-derived frontal and paraxial mesoderm-derived parietal bone and subsequently the role of specific signaling pathways in determining apoptotic activity. TGF-β, BMP and Wnt have previously been shown to play important roles as apoptosis regulatory signaling pathways and so our attention was first drawn to these pathways [116–125]. Our recent study [126], demonstrates significant differences in caspase-3 activity between FOb and POb cells when undergoing osteogenic differentiation, with elevated levels in POb compared to FOb cells. Interestingly, TGF-β activity as analyzed by endogenous phosphorylation of Smad2 revealed higher activation of this pathway in POb cells. TGF-β signaling, therefore, represented the first signaling pathway to be more activated in POb, a reciprocal activity profile to that of FGF and Wnt signaling pathways, as previously outlined [11,45,46]. This observed profile for TGF-β was present before and after commencement of differentiation. Conversely, blocking this pathway with the specific inhibitor SB431542 was shown to reduce apoptosis and improve osteogenic capacity of mesodermal derived POb cells. Secondly, we observed enhanced activation of BMP signaling in FOb compared to POb and a potential role in protecting FOb from excessive apoptosis. Treatment with noggin, a potent BMP signaling inhibitor, increased apoptosis in FOb while exogenous BMP-2 decreased apoptosis in POb cells. We also identified that inhibition of the Wnt signaling pathway, with Dkk1 and sFRP, dramatically increased apoptosis in cells, while decreasing osteogenic differentiation. Stimulation with Wnt3s had the opposite effect of decreasing apoptosis. Our *in vitro* data therefore provides a useful insight into the way multiple signaling pathways can integrate to coordinate physiological processes such as apoptosis and osteogenesis (Figure 2). Regional differences in apoptotic activity will ultimately determine the number of osteoblasts at any given time in relation to the number of osteoclasts, and therefore will affect the rate of bone formation and regenerative capacity. Importantly, these data are in keeping with our previously published work that FOb cells possess a greater osteogenic potential than POb cells and confer a greater regenerative capacity on neural crest derived frontal bones [11,45,46].

## 6. Conclusions

It is clear then that the multiple ancient and highly conserved signaling pathways that govern osteogenesis and the endogenous regenerative capacity of calvarial bones interact in a myriad of ways. Furthermore, another dimension of complexity is added when one considers that they may interact differently depending on context, intensity, duration and spatiotemporal timing of signaling activity. Nevertheless, despite this complexity, striving for a deeper understanding of the multiple signaling pathways, which constitute a putative osteoblast regulatory network, and the intricacies of their integration, is a worthwhile cause. It will allow us to improve the effectiveness with which we can enhance endogenous calvarial healing by identifying key components of this network as suitable targets for selective pharmacological modulation, and thereby potentially provide the most appropriate reconstructive solution of all for craniofacial reconstruction—endogenous calvarial regeneration. This would obviate the need for contemporary surgical approaches, which carry significant deficiencies. Furthermore, of unique clinical importance is that adopting a solely pharmacological strategy for bone regeneration without the reliance on cell-based therapies would avoid the perceived risks of such therapies such as tumorigenicity, the risk of transmitting infection and genetic disease, the risk of contamination or cellular damage, and concerns regarding cellular purity. Such an approach is therefore likely to be more readily acceptable to the FDA and translatable into the clinical arena.

Evolution has bestowed a superior regenerative capacity on neural crest derived frontal bone. The multiple signaling pathways and the complexity of their integration in time and space is implicit in conferring this evolutionary regenerative advantage. Therefore, by investigating these regional embryonically determined differences, we believe it will be possible to unravel some of the hitherto undiscovered aspects of cell signaling in bone biology.
